# Immunotoxins Immunotherapy against Hepatocellular Carcinoma: A Promising Prospect

**DOI:** 10.3390/toxins13100719

**Published:** 2021-10-11

**Authors:** Mohammad Heiat, Hamid Hashemi Yeganeh, Seyed Moayed Alavian, Ehsan Rezaie

**Affiliations:** 1 Baqiyatallah Research Center for Gastroenterology and Liver Disease, Baqiyatallah University of Medical Science, Tehran 1435916471, Iran; mohamad.heiat@gmail.com (M.H.); alavian@thc.ir (S.M.A.); 2 Department of Biochemistry, Faculty of Biological Science, Tarbiat Modares University, Tehran 14115111, Iran; hamid_hashemi001@yahoo.com; 3 Molecular Biology Research Center, Systems Biology and Poisonings Institute, Baqiyatallah University of Medical Science, Tehran 1435916471, Iran

**Keywords:** immunotoxin, hepatocellular carcinoma, targeted therapy, liver cancer, drug targeting

## Abstract

Hepatocellular carcinoma (HCC) is one of the most common cancers in the world. Therefore, fighting against such cancer is reasonable. Chemotherapy drugs are sometimes inefficient and often accompanied by undesirable side effects for patients. On the other hand, the emergence of chemoresistant HCC emphasizes the need for a new high-efficiency treatment strategy. Immunotoxins are armed and rigorous targeting agents that can purposefully kill cancer cells. Unlike traditional chemotherapeutics, immunotoxins because of targeted toxicity, insignificant cross-resistance, easy production, and other favorable properties can be ideal candidates against HCC. In this review, the characteristics of proper HCC-specific biomarkers for immunotoxin targeting were dissected. After that, the first to last immunotoxins developed for the treatment of liver cancer were discussed. So, by reviewing the strengths and weaknesses of these immunotoxins, we attempted to provide keynotes for designing an optimal immunotoxin against HCC.

## 1. Introduction

HCC is the sixth common type of malignancy worldwide and also the most common form of primary liver cancer [1,2]. There are three types of primary liver cancer: two main and one rare. HCC and cholangiocarcinoma are known as the most common types of primary liver cancer. The last primary liver cancer, known as angiosarcoma, is a rare type of liver cancer. HCC is a flagship of cancer-related death all over the world. Based on the SEER database, 65% of all cases of liver cancers belong to HCC [3]. According to a worldwide estimation of cancer incidence in 2008, HCC involved males 2.4 times more than females [4] and is the 5th and 7th most common cancer in males and females, respectively. Therefore, high percentages of individuals (around 85%) with HCC are suffering from cirrhosis [5]. Patients with cirrhosis caused by HBV, HCV, and even non-cirrhotic carriers of HBV, and those with chronic HCV are at risk for HCC. As a result of several reasons, HCC has a poor prognosis with a 6.9% five-year survival rate [6].

Chemotherapy drugs, as well as radiation therapy, act in a non-specific manner, destroying all exposed tissues and have destructive effects on normal cells [4]. On the other hand, some cancers (HCC, hematological malignancies, leukemia, lymphoma, and Hodgkin’s disease) are resistant to common treatments such as chemotherapy and radiotherapy in the first stage and also after one or more relapses [7]. For reducing many side effects of common treatments, the targeted therapies, including monoclonal antibodies such as anti PD-1 mAb [8] or small-molecule drugs such as miRs [9], have attracted a lot of interest among researchers. The recombinant immunotoxins are armed mAbs with a toxic moiety to earn higher cytotoxicity against the cancer cells. Immunotoxins are highly effective and potent agents in cancer therapy, so that one immunotoxin molecule can kill a targeted cancer cell alone.

The global market for cancer therapeutics is anticipated to reach €240 billion in 2023, and more than 60 immunotoxins are currently undergoing pre-clinical and clinical testing [10]. Between 2013 and 2017, the number immunotoxins in the clinical trials was tripled [11]. They have been effective in treating several types of cancers. The Denileukin Diftitox (ONTAK) as a ligand–toxin drug was approved by the FDA in 1999 for the treatment of cutaneous T cell lymphoma [12], and in 2014, the marketing of Ontak was discontinued in the US. Moxetumomab Pasudotox was also approved by the FDA in 2018 and is now applied against hairy cell leukemia [13]. So far, many immunotoxins have been introduced to treat HCC. In this review, after defining the immunotoxin and describing its mechanism of action, the factors affecting the function of immunotoxins are reviewed with an emphasis on liver cancer. Moreover, for the first time, the specific markers on liver cancer cells, focusing on treatment with immunotoxins, are introduced. Thenceforth, all immunotoxins developed to treat liver cancer are listed and discussed. This way, the present review can help the selection of appropriate liver tumor markers and the design of an effective immunotoxin against liver cancer cells in the future.

## 2. Immunotoxins

Immunotoxins (as comes from their name) are constructs containing a monoclonal antibody (mAb)/part of mAb and fully or truncated forms of a potent cytotoxin chemically or genetically bound together. So, the potent toxins are raised as key agents in cancer treatment as well as in protection against infectious agent [14]. *Pseudomonas* exotoxin A (PE) is used in the structure of many immunotoxins. There are some advantages in the PE structure over other plant or animal toxins that lead to using PE as a dominant toxin in the immunotoxins structure. Over time, PE was evolved in terms of structure and function especially in view of binding, processing, routing, and toxicity [15]. This toxin, unlike other toxins, has a KDEL-like sequence at the C-terminus for the retrograde transportation to the endoplasmic reticulum (ER). PE also has a furin-cleavable motif in domain II (aa 274–280, RHRQPRG) that with a conformational change makes it accessible. This furin-cleavable motif is necessary for further detaching a 37 kDa fragment of PE that includes domain II, domain Ib, and domain III with ADPribosylase activity [16]. It seems that the PE structurally enables us to manipulate and remove its immunogenic epitopes so that far less immunogenic PE with deletion or mutation in its B and T cell immune-dominant epitopes has been obtained [17]. This toxin is formed by a Ia fragment (as the binding domain), Ib and II fragments (as the translocation domains) and a III fragment (as the catalytic domain). To design a PE-based immunotoxin, the binding domain is replaced by a ligand or part of a monoclonal antibody.

Most of the original immunotoxins were constructed by using murine antibody or antibody fragments. The murine antibodies may stimulate human antibodies in response to immunogenic murine regions (HAMA) or may stimulate the production of anti-drug antibodies (ADA). Therapeutic monoclonal antibodies with framework humanization, chimerization, and the use of mice with humanized germlines can be engineered to dramatically decrease their immunogenicity. However, there is 9% immunogenicity against variable complementarity-determining region domains even for humanized antibodies [18]. The single-chain Fv fragments are composed of light- and heavy-chain variable domains and are the smallest binding unit of an antibody; using this fragment, the efficiency of its penetration into the cancer cells is increased [19]. So, the fully humanized scFv with higher penetration and less immunogenicity is the best option for immunotoxin designing [20]. The most commonly used linker in immunotoxins is repetitions of EAAAK or GGGGS (as flexible and rigid likers, respectively) [21]. In addition, the dual- or multi-functional linkers are designed and applied in immunotoxin structures for different purposes. For example, the “EAAAKECCPGCCMEP” linker has been introduced as a linker that functions in both immunotoxin’s imaging and steric hindrance prevention [22]. The selected toxin can be a bacterial toxin such as *Pseudomonas* exotoxin, diphtheria toxin, or shiga toxin and also can be a plant toxin such as ricin, saporin, and gelonin. The initial immunotoxin studies on hepatoma cells were based on plant toxins including trichosanthin and gelonin toxins in 1991 and 1994, respectively [23,24]. These studies gave promising results about the potent and quite specific anti-hepatoma agents. There are many advantages of using immunotoxins in comparison with other therapeutic approaches, including fewer side effects, a simple preparation method, and low production cost. Furthermore, one of the immunotoxins’ advantages is that they can be easily expressed in microbial hosts. For the use of immunotoxins in the clinic, there are some unsolved challenges yet. One of the basic problems with immunotoxins is their immunogenicity. Therefore, the protein nature of immunotoxins leads to the immune reaction and production of ADA. This event can be followed by immune-related adverse events, including infusion-related reactions, allergic or anaphylactic reactions, delayed hypersensitivity, and autoimmunity [25]. Vascular leak syndrome (VLS) is another major side effect of immunotoxins in which it increases vascular permeability with the extravasation of fluids and proteins, resulting in interstitial edema and in severe cases pulmonary and cardiovascular failure [26]. This occurs due to having the one set of motifs that makes it possible for an immunotoxin to attach to the epithelial cells surrounding the blood vessels, and it causes dose-limiting toxicities. Hepatotoxicity can be accounted as the other side effect that has been terminated some clinical trials. However, to overcome ADA, VLS and hepatotoxicity presented some promising strategies including decreasing the immunogenicity of immunotoxins by deletion/mutations in the B or T cells’ epitopes, deletion/mutations in the VLS-agents motifs, and using humanizing antibodies.

**Figure 1:**
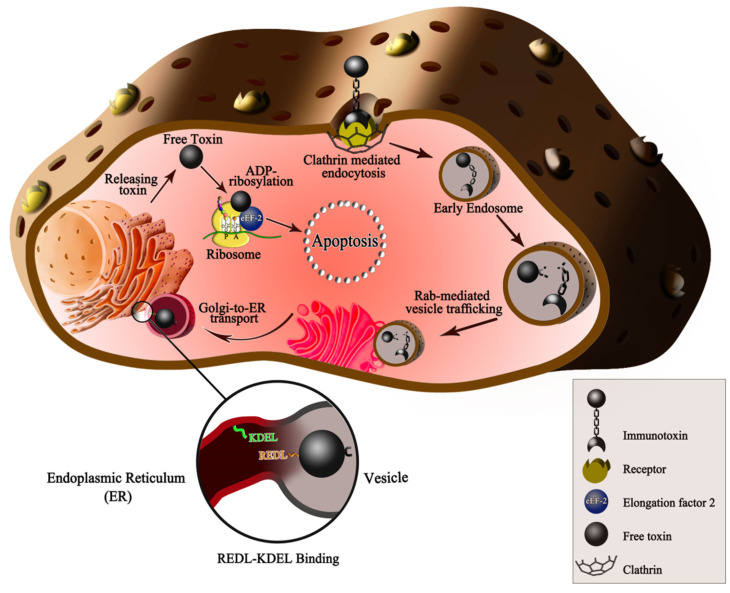
The immunotoxin’s mechanism of action on hepatocellular cancer cells. After the ligand binding to receptors in clathrin-coated pits, receptor-mediated endocytosis begins, and then, the budding of the pits. In the early endosomal environment, the 37 kd toxin moiety of PE is separated. This process is related to the conformational change following exposure of the furin-cleavable motif (amino acids 274–280 from II domain, RHRQPRG). In the late endosome, the 37 kd toxin moiety uses Rab proteins to reach the trans-Golgi network (TGN). The toxin moiety of PE has an REDL motif (amino acids 609–612) in the C-terminus, which can bind to the KDEL receptor (KDEL R: green line in the picture) on the ER. Changing the C terminal RDEL to the KDEL motif is one modification on the PE in which the binding capacity to KDEL R has been improved. Thus, the toxin can be transported to the ER and then to the cytosol in a retrograde manner. In the cytosol, the 37 kd toxin moiety begins its enzymatic activity and causes ADP ribosylation in the elongation factor-2 (eEF-2) on the ribosomes. Accordingly, the eEF-2 is inactivated, and the protein synthesis of the target cells is inhibited, resulting in irreversible apoptosis induction.

PE toxin as a model is considered to see the immunotoxin’s mechanism of action. Some tumor markers have high expression in HCC. These cells have different endocytic mechanisms and entry routes for selective internalization. These routes include receptor-mediated endocytosis, lipid raft-associated endocytosis, caveolae, or fluid-phase uptake [27]. The immunotoxins are usually entered into the cell through the clathrin-dependent endocytosis pathway. The HCC-specific immunotoxins can bind and then internalize to cancer cells by the targeting moiety. After internalization, the toxic moiety, in a retrograde manner, can reach the target molecule in the cytoplasm and causes apoptosis by protein synthesis inhibition or other actions (Figure 1). The PE intoxication happens in a short time. In the previous studies about the intoxication of PE into rat liver, it was shown that the PE binding and association happened after 5–30 min, endosome forming after 15–60 min, and finally PE translocation into the cytosol after 30–90 min [28]. In the first immunotoxin structures, full-length PE was conjugated with whole mAbs or receptor ligands. Over time, domain Ia of PE was replaced by a ligand or the variable fragment (Fv) of a mAb and formed PE40. For decreeing immunogenicity, another version of PE, namely PE38, was designed in which the majority of domain Ia (Δ1–250) and a portion of domain Ib (Δ365–380) was deleted. The next modification in the PE was the replacement of REDLK native sequence located in the C-terminal amino acids 609–613 with KDEL sequence. This signal sequence enhances the cytotoxicity of PE-based immunotoxin by improving the efficiency of Golgi to ER transport [29].

## 3. Factors Influencing Immunotoxins Efficiency in HCC

There are many factors affecting immunotoxins efficiency, including the type, localization and rate of expression of targets, internalization procedure, etc. As mentioned previously, the immunotoxins contain a binding moiety that can target only cell surface markers. Consequently, it is logical that the first issue to antigen targeting with an immunotoxin is the antigen expression on the cell surface. Secondly, because normal cells may express the target antigens in a low concentration and get affected by immunotoxins [30], the rate of antigen expression on the target cells has a significant role in the drug effectiveness [31]. The Her-2-targeted immunotoxin erb-38 has stopped in a phase I clinical trial because of all of the first six patients having significant hepatotoxicity [32]. Further investigations identified very low-level Her-2 expression on the surface of hepatocytes that had not been appreciated in prior studies.

The antigens selected for antitumor targeting should be tumor-associated antigens, with either a tumor-specific or a differentiation pattern of expression on cancer cells [33]. These antigens include viral antigens, antigens encoded by cancer-germline genes, antigens encoded by mutated genes, differentiation antigens, and overexpressed antigens [34]. Antigens encoded by mutated Ras proto-oncogenes genes have been observed in HCC. The N-Ras codon 61 [34], the H-Ras codon 12 [35], and the K-Ras codon 12 mutation [35] are the most commonly investigated mutations in HCC. It was noted that most of the mutated antigens and viral antigens are localized to the cytosol and thus, they are not good markers for targeting by immunotoxins. The overexpressed antigens are more interesting because of their cell surface expression.

After immunotoxin binding to its target on the cancer cells, the internalization procedure is accentuated for the immunotoxin efficacy and potency. It is suggested that the immunotoxin potency and cytotoxicity have significant correlation with the internalization rate into the cells [36]. For example, there are two targeted toxins with names DT388IL3 [37] and 26,292 (Fv)-PE38KDEL [38] against an IL3 Receptor (CD123). DT388IL3 is composed of diphtheria toxin (DT388 including the translocation and catalytic domains) linked to the interleukin 3 (IL3) but 26,292 (Fv)-PE38KDEL is composed of single-chain Fvs fused to a 38 kd fragment of Pseudomonas exotoxin A. Despite the difference in the type of toxin and the binding moiety, these two targeted toxins are similar in the receptor and the targeted cancer, interleukin-3 receptor (IL-3Ralpha, CD123) and acute myeloid leukemia (AML), respectively. Although the 26,292 (Fv)-PE38KDEL like DT388IL3 has a strict binding to CD123, it has 50-fold lower cytotoxicity [39].

Antigen-binding affinity, epitope location, amino acid types on the antibody paratopes, and the rate of target antigens on the cell surface are many other factors that can affect the immunotoxin efficiency [40,41].

## 4. HCC Tumor Markers

The recognition of tumor markers is necessary for the development of targeted therapy approaches [42,43]. Here, HCC tumor markers are presented, some of which are used in immunotoxin immunotherapy, and some of them could be good candidates in this regard (Table 1). Response to therapy in a targeted tumor marker has depended on its expression patterns in liver cancer tissues, and it can be different in patients with the same cancer. One type of good candidate markers for targeted therapy is onco-fetal antigens that are overexpressed during fetal development and transcriptionally silenced after birth [44]. These antigens are not expressed on the normal cells, while the tumor cells re-express them. Table 1 includes one member of this antigen group, glypican 3 [45]. However, the overexpressed normal antigens are the largest category of antigens that can be targeted with a monoclonal antibody or immunotoxin. This category is characterized by great overexpression compared to its levels in the normal cells [44]. The epithelial cell adhesion molecule (EPCAM), epidermal growth factor receptor (EGFR), fibroblast growth factor receptor (FGFR), mucin core protein 1 (MUC1), etc. are in this category.

Some disadvantages limit the efficiency of overexpressed antigens for applying as a target on hepatocyte cancer cells. For example, E-cadherin as an epithelial marker, by decreasing its expression in epithelial mesenchymal transition (EMT) and re-overexpression in secondary metastatic cancer cells, plays some role in epithelial cell–cell adhesion [46,47]. In contrast to E-cadherin, β-catenin is a mesenchymal marker with increased expression in both metastatic cancer cells and EMT [48]. EPCAM, EGFR, and MUC1 glycoproteins are overexpressed only on the surface of epithelial cancer cells, not on cancer cells with mesodermal and ectodermal origins. In addition, EPCAM is overexpressed on normal cells in inflammatory diseases as well [48]. Monoclonal antibodies against MUC1 may inhibit metastasis [49]. It is also reported that the glycosylation pattern of MUC1 is different in normal and cancer cells, which is a positive point to be used for designing specific antibodies. EGFR, FGFR, and PDGFR have crucial roles in EMT and metastasis [50,51]. E-cadherin leads to FGFR’s aberrant endocytosis in endothelial cancer cells. Before metastasis and in primary tumors, E-cadherin expression increases, which in turn increases cell–cell adhesion and decreases the endocytosis of FGFR [52]. During metastasis, the expression and internalization of FGFR enhances following decreased E-cadherin expression [53]. Therefore, antigens such as β-catenin, MUC1, EGFR, FGFR, and PDGFR are sufficient targeted therapies for metastatic cancer cells, while E-cadherin is efficient for cancers in primary stages or after metastasis. It is noticeable that EGFR, FGFR, and PDGFR are very active, even in endosomes following endocytosis, making signals for cell survival and progression. Therefore, the toxicity of immunotoxins must be high enough to kill cancer cells before signaling onset [54]. CD40 is a 45–50 kD glycoprotein expressed on the surface of B-lymphocytes and dendrite cells as well as cancer cells. CD40 has a dual function in cancer promotion and immune system activation such as APC, T-lymphocytes, and macrophages to kill cancer cells. Monoclonal antibodies targeting CD40 activate lymphocytes to produce more natural antibodies against CD40 to increase recognition by the immune system [55]. However, recent studies showed that targeting CD40 using monoclonal antibodies decreased the number of platelets, monocytes, and lymphocytes [56]. Among the CXCRs family, CXCR7 possesses crucial roles in proliferation, cell survival, metastasis, and angiogenesis. As a result of ensconcing CXCR7 in the cytosol of normal cells such as neuronal and astroglial cells, it is not detectable in normal cells, whereas it is overexpressed dramatically on the surface of cancer cells. By this, antibodies against CXCR7 exhibit largely specific behavior [57]. Once, targeting and blocking CXCR7 inhibited metastasis [58]. A high concentration of chemokine has a negative effect on CXCR7 expression but no influence on internalization. Generally, the rate of CXCR7 internalization is independent of the binding ligand [59]. The mesothelin receptor is expressed on mesothelium cells and overexpressed on cancer cells, which participate in metastasis and chemotherapy resistance [60]. Studies revealed that targeting this receptor using monoclonal antibody and SS1P immunotoxin (PE38-Ab) had the appropriate function to inhibit cancers [61,62]. TRAILRs and TNFRSF, which are overexpressed in cancers, play roles in apoptosis. TRAILR1 and 2 contain the death domain, whereas this domain is absent in TRAILR3 and 4 and acts as a decoy receptor to regulate apoptotic function. Injecting extrinsic recombinant TRAIL as a ligand or monoclonal antibody for TRAILR1 and 2 induced apoptosis in cancer cells without adverse effects on normal cells [63]. TNFRs were expressed on T-lymphocytes and APCs to produce natural antibodies against cancer cells antigens. Therefore, in addition to targeting cancer cells, the immune system is exposed to targeting moieties. By this, targeting such antigens with an antibody or ligand can have fewer adverse effects than an immunotoxin [64]. Although TNFRs induce apoptosis, they have roles in cell survival, proliferation, invasion, and angiogenesis [65]. The asialoglycoprotein receptor (ASGPR), a high-capacity C-type lectin receptor, is frequently expressed on hepatocytes (500,000 ASGPR/hepatocyte) and minimally expressed on other tissues. The physiological function of this receptor is the specific binding and internalization of galactose (Gal), and because of this, it is frequently used for hepatic delivery [66]. In addition, this receptor was previously used as a target for immunotoxin. The p230 is a 230 kDa membrane-bound protein that is expressed in several types of cancers including HCC, melanoma, and breast cancer. Receptor protein p230 was firstly discovered in melanoma and is applied for melanoma diagnosis with immunohistochemical evaluations. The SM5-1 monoclonal antibody can bind specifically to p230 overexpressing HCC cancer. To increase the efficacy of SM5-1 on HCC, it conjugated with an Au nanoparticle (Au-SM5-1) and evaluated its effects on tumor cell proliferation, angiogenesis, and apoptosis [67].

Gamma-Glutamyltransferase (GGT) is a surface enzyme of kidney tubules, brain capillaries, and biliary epithelium with a role in redox regulation. Overexpressed GGT in cancer cells increases proliferation, invasion, and chemotherapy resistance [68]. GGT can contribute to the cellular antioxidant process and increase cellular tolerance against oxidative stress. GGT inhibited with many chemotherapeutics compositions, including glutamine analogs acivicin (AT125), 6-diazo-5-oxo-L-norleucine, azaserine, boronate derivatives, L-glutamic acid derivatives, gamma-monophenyl, and phosphonoglutamate analogs, but finding the less toxic drugs remains an interesting perspective of pharmacological research [69]. So, GGT as a key marker on liver [70] and also by its role in the tumor progression, invasion, and drug resistance can be a good marker to immunotoxin immunotherapy. HIF-1a is a transcription factor that keeps cancer cells alive under hypoxic conditions through switching from the most efficient to lowest energy production to maintain the level of energy production continuously and/or angiogenesis [71]. As a result of being in the nucleus, HIF-1a is not appropriate for targeting by immunotoxin. Frizzled receptors (FZD) interfere with Wnt signaling and cancer cell progression. In addition to cancer cells, some Frizzled receptors are expressed in normal cells but at a lower level [72]. The mRNA expression profiles related to FZD2 and 7 showed their overexpression in late stages of primary liver and lung cancers compared with normal tissue and early-stage cancer [73]. A monoclonal antibody, known as OMP-18R5, was generated against the conserved epitope of extracellular domain of FZD1, 2, 5, 7, and functionally causes a reduced growth of human tumor xenografts in mice [72]. An immunotoxin-based therapeutic strategy against the overexpressed FZD receptors in HCC such as FZD2 and 7 could be a translatable approach to overcome cancer.

In general, the antigens and receptors expressed on the cell surface that play a role in the signaling pathways should be considered as targets for ligands, monoclonal antibodies, and immunotoxin targeting. In conclusion, the antigens expressed in both normal and cancer cells with a low differential expression pattern are better to be used as targets for monoclonal antibodies or their ligands, and those overexpressed on cancer cells can be utilized as targets for immunotoxin recognition. As a result, blocking antigens using monoclonal antibodies (which may contain toxin moiety) or inhibitors inhibit tumor progression. Immunotoxins against antigens not only have the potential to block signaling pathways by preventing ligand binding to antigens but also have toxic effects on cancer cells after internalization.

## 5. Anti-HCC Immunotoxins

**Figure 2:**
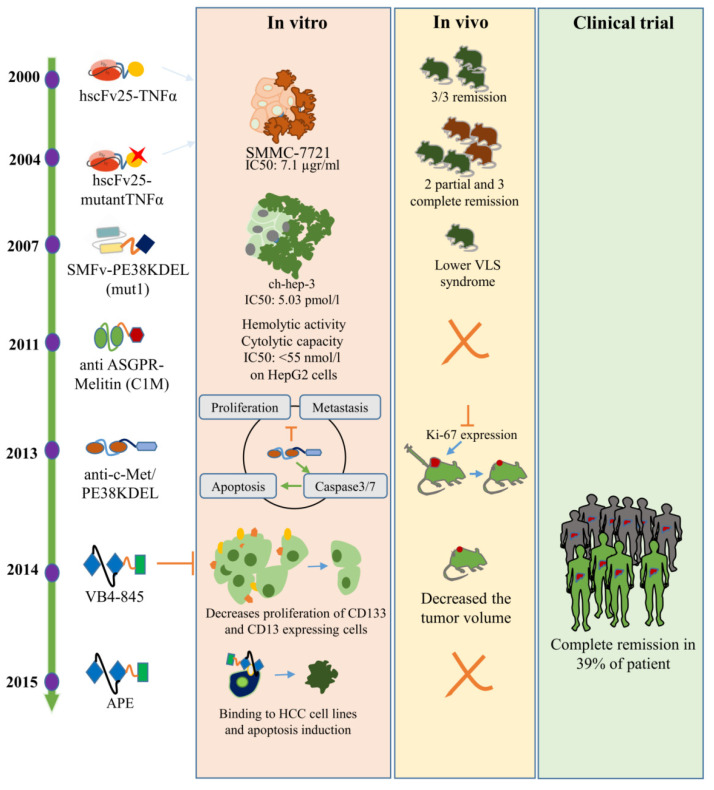
HCC-specific immunotoxins since 2000 to 2015. In vitro, in vivo, and clinical trial results related to introduced immunotoxins are shown in pink, orange, and green background, respectively.

The schedule of immunotoxin studies on HCC began in 2000 (Table 2 and Figure 2). Sun et al. (2000) launched the first serious practice for the application of immunotoxins against HCC in a nude mice model with HCC xenograft. In order to generate a bioactive immunotoxin, they linked hscFv25 (humanized anti-human HCC single chain fragment) with the human TNFα gene. The hscFv25-TNFalpha inhibited the growth of the human hepatocarcinoma cell line SMMC-7721 with an IC_50_ of was 7.1 µgr/mL. Their findings obtained from a tumor regression trial showed that the efficiency of the hscFv25-TNF-alpha was more than that of TNF-alpha (3/3 vs. 2/3 remission) [89]. Four years later, in the same laboratory, Zhang et al. (2004) produced a fusion immunotoxin by designing a recombinant construction carrying the genes coding for mutant human TNF-alpha and human scFv. The toxicity of mutant human TNF-alpha (with the deletion of seven amino acids from N-terminus and Pro8 Ser9 Asp10 changed to Arg Lys Arg, and Leu157 changed to Phe from the C-terminus) decreased and its cytotoxicity increased compared with natural TNF-alpha. Treating with such immunotoxin, they showed partial and complete remission in 2/5 and 3/5 of HCC model mice, respectively. It is noteworthy that the minor response was obtained when treating by mTNF-alpha alone (5/5 partial remission), but no remission was observed in the placebo group. However, due to the few numbers of nude mice preventing rigorous statistical significance and more experimental investigations needed, the hscFv25-mTNF-alpha showed a better distributive ratio in HCC tissues. Their findings determined that immunotoxins, even in a general mode, could be considered as an interesting drug to overcome HCC [90]. 

Wang et al. (2007) focused on HCC cells overexpressing a membrane protein (p230) [91]. Regarding vascular leak syndrome (VLS) as one of the most serious obstacles in the application of immunotoxins, they created a high specific fusion immunotoxin through genetically fusing a derivative of anti-p230 antibody (SM5-1 monoclonal antibody) with PE38KDEL. Eight mutant immunotoxins were designed to reduce VLS side effects. Among them, mut 1 (R318K, N441Q, R601K) had more efficiency. They declared that this recombinant mut 1 IT (with IC50 5.o3 and 0.2 pM for ch-hep-3 and ch-hep-1, respectively) specifically killed tumor cells in mice, with a weak induction of VLS. Moreover, using the KDEL motif instead of EDLK in this immunotoxin probably has been affective in its high cytotoxicity, as shown in our previous studies on PE38 with [92,93] or without [94] KDEL motif-based immunotoxin. The authors suggested that mut 1 could be presented as a novel and effective candidate of PE-based immunotoxin for use against SM5-1 binding positive cells such as HCC cells.

To decrease the immune response against the toxin moiety (PE) of mut 1, they produced PE38KDEL-I-loaded nanoparticles conjugated with F(ab’) fragments of a humanized SM5-1 (PE-NP-S) [91]. Their assessments confirmed that PE-NP-S had much more promising attributes including increased cancer therapeutic efficacy, weaker induction of VLS in mice, higher LD_50_, lower immunogenicity, and less sensitivity to inactivation by anti-PE neutralizing antibodies than mut 1.

In direction of this type of targeted therapy, researchers have always sought to produce the best-performing immunotoxins, so they have not only altered the composition of recombinant immunotoxins but also assessed the functional potential of different targets. In this way, Zhao et al. (2011) constructed a recombinant IT by fusing melittin with scFv of the anti-asialoglycoprotein receptor (ASGPR) denoted as C1M. They confirmed the hemolytic activity and cytolytic capacity of C1M, which was mediated by melittin. The melittin is a very non-specific cytotoxic peptide that attacks all lipids on the cell membrane, resulting in plasma leakage and cell death. Conjugating melittin with scFv of ASGPR leads to killing specifically ASGPR-positive tumor cells. According to their analysis, a lack of C1M effect in coadministration with asialoorosomucoid (ASGPR ligand) confirmed its target-specific cytotoxicity [100].

In another work, Liu et al. in 2013 evaluated a recombinant fused IT consisting of anti-c-Met ScFv and a modified pseudomonas exotoxin A (PE38KDEL) against HCC [96]. The c-Met (an overexpressed receptor tyrosine kinase in a broad range of human malignancies) was targeted as an ideal gateway for IT therapy. In vitro analysis revealed that their recombinant IT known as anti-c-Met/PE38KDEL specifically bound to c-Met-positive hHCC cell lines and made defects in cell proliferation through caspase-3/8-mediated apoptosis. It was suggested that anti-c-Met/PE38KDEL might prevent metastasis [101]. The in vivo investigations also demonstrated that the administration of this IT led to increased rates of tumor cell apoptosis and suppression of the Ki-67 expression, causing inhibition in the growth of HCC xenografts. 

Epithelial cell adhesion molecule (EpCAM), a prognostic predictor and progenitor HCC tumor marker, was the subject of the study of Ogawa et al. They tried to evaluate the in vitro and in vivo effects of immunotoxin VB4-845 on EpCAM-positive HCC cells. Already, the VB4-845 immunotoxin had been developed by Viventia Biotech Inc. in 2008 for treating bladder (undergoing phase II clinical trial) and head and neck cancers (undergoing phase II and III clinical trials) [102]. In evaluations, VB4-845 introduced itself as a potent cytotoxic IT for all EpCAM expressing HCC cells by suppressing the sphere formation in HCC. The stemness, which is the expression of stem/progenitor markers such as EpCAM, CD133, CD13, CD44, and CD90, has the potential for self-renewal, which derives tumorigenesis. It is shown that the VB4-845 forcefully decreases the CD133 and CD13 expression, suggesting that VB4-845 may affect the stemness [97]. Immunotoxin VB4-845 was in the phase I trial. Complete remission was observed in 39% of patients after treatment without negative side effects [103]. In 2014, another immunotoxin named pachyerosin-SM0736 with conjugation of pachyerosin as a plant toxin with anti-human AFP monoclonal antibodies SM0736 was developed that showed ability in the inhibition of growth on human hepatoma cell line HuH-7 [104].

One year later, in 2015, Chinese researchers again targeted EpCAM by another recombinant immunotoxin [20]. They genetically constructed an immunotoxin denoted APE through fusing a truncated toxin (PE38KDEL) with a single-chain variable fragment (scFv) of an anti-EpCAM. The results of ELISA, flowhahahha cytometry, and MTT assay confirmed the binding and cytotoxic traits of APE on EpCAM-positive HCC cells. They suggested further investigation including testing in animal and immunotoxin modification to decrease immunogenicity.

**Figure 3:**
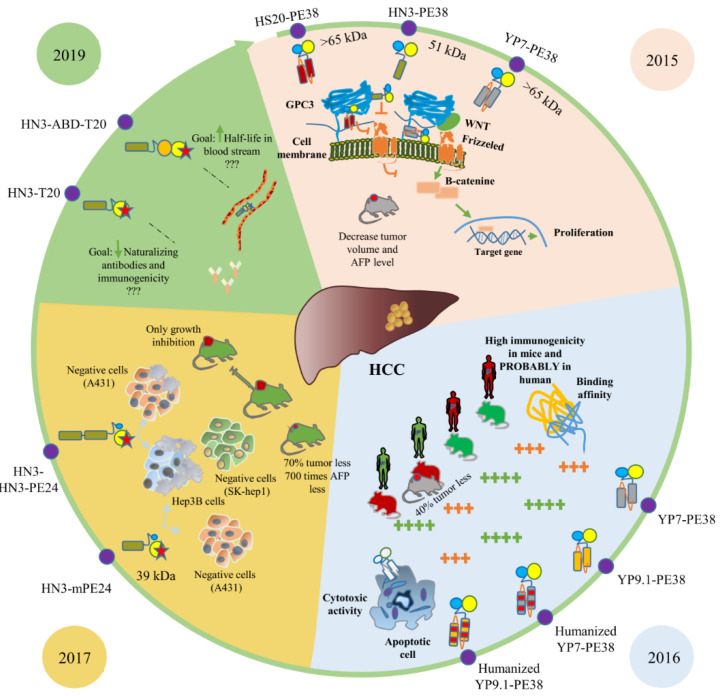
GPC3-targeted immunotoxins. Immunotoxins that target the GPC3 receptor were introduced from 2015 to now. Those studied by the same research group in the same year were compared together and presented in an identical background stain.

The identification and evaluation of glypican 3 (GPC3) have revolutionized the research process of HCC immunotherapy (Figure 3). As a cell-surface proteoglycan, GPC3 is overexpressed in human HCC cells and absent from normal tissues. For the first time in 2015, Gao et al. developed three antibodies (HN3, HS20, and YP7) targeting glypican 3 [5]. HN3 and HS20 inhibited the Wnt signaling pathway by blocking a functional epitope of GP3 and YP7 just specifically bound to the C-terminal epitope without any biological function. These described that antibodies in conjugation with pseudomonas exotoxin A (PE38) respectively served as targeting and toxic moieties of two fusion immunotoxins. Both in vitro and in vivo results revealed that HN3-PE38 possessed a superior antitumor function in comparison with YP7-PE38 and presented itself as a potent candidate for immunotoxin-based HCC therapy. Of course, it should be considered that perhaps the promising results of HN3-PE38 and HS20-PE38 raised from dual traits including the blocking of cancerous cell signaling mediated by antibody and suppression of protein synthesis by toxin moiety, whereas YP7-PE38 acts just as a protein synthesis inhibitor. According to this, HN3-PE38 and HS20-PE38 are more effective for tumor suppression compared with YP7-PE38.

In 2016, Zhang et al. designed humanized anti-GPC3 scFvs, namely hYP7 and hYP9.1 fused with PE, and investigated their affinity and cytotoxicity. They grafted the combined KABAT/IMGT complementarity determining regions (CDRs) of mouse scFvs into a human IgG germline framework. In spite of a very similar scFv sequence of mouse YP7-, YP8-, YP9-, and YP9.1-PE38 immunotoxins, YP7-PE38 and YP9.1-PE38 showed higher efficiency and performance, and subsequently, they were chosen to be humanized. In vitro and in vivo analyses revealed that mouse YP9.1-PE38 had better binding affinity and cytotoxicity compared with mouse YP7-PE38. In humanized immunotoxins, the binding affinity of hYP9.1-PE38 was better, but hYP7-PE38 was more cytotoxic. In vivo study indicated the antitumor activity of hYP7-PE38 in nude mice. The high performance of immunotoxins containing mouse scFvs refers to the generation of antibodies against humanized ScFv in mice. Although clinical trials are not performed for immunotoxins with humanized scFv, it seems that this humanization has positive effects on the reduction of immunogenicity [98].

Studies on this biomarker continued in 2017 by Wang et al. [48]. They made efforts to design and construct two recombinant ITs against HCC cells, including a modified truncation of *Pseudomonas* exotoxin A (mPE24) and HN3 human single-domain VH as the targeting anti-GPC3 antibody in one and two repeats (HN3-mPE24 and HN3-HN3-mPE24). Their evaluation of HN3-mPE24′s antitumor activity in the mice model revealed that the level of alpha-fetoprotein in the treated group was 700-fold lower than in the untreated group. Furthermore, at the end of their research (15 weeks after tumor implantation), 25% of the treated mice were still alive. The high cytotoxicity of HN3-PE38 is caused by its small size and rapid penetration into tumor tissues in vivo. Although small immunotoxins have the potential to be removed from blood serum rapidly, a low-dose, high-frequency treatment might be effective. The HN3-HN3-mPE24 has a bivalent binding moiety that enables it to increase binding affinity compared with HN3-mPE24. The increased size of HN3-HN3-mPE24 resulted in enhancing its half-life in mice serum and decreased penetration in a xenograft tumor. Significant tumor suppression and increased survival without off-target effects after treating mice with HCC suggests that the HN3-mPE24 can be a promising agent against HCC. Despite all benefits of HN3-mPE24 as a good candidate for HCC treatment, because of the non-specific cytotoxicity related to higher doses, it requires re-engineering for clinical trials [48].

Immunogenicity and a short serum half-life limit the application of immunotoxins. To overcome this challenge, Mitchell et al. [99] engineered two new immunotoxins, HN3-T20 and HN3-ABD-T20. In HN3-T20, the HN3 is a binding moiety fused to mutant PE toxin that was modified to remove domain II and six T cell epitopes [105]. To enhance HN3-T20′s serum half-life, they added a streptococcal albumin-binding domain (ABD) and a llama single-domain antibody fragment specific for mouse and human albumin to HN3-T20. Adding ABD caused a 45-fold higher serum half-life than HN3-T20 (326 min vs. 7.3 min). Both HN3 and HN20 human monoclonal antibodies were designed to target the Wnt binding motif on the GPC3 protein and were able to block the effects on Wnt signaling, resulting in HCC tumor growth inhibition in vitro and in vivo. Another human monoclonal antibody against GPC3, named 32A9, was designed to recognize an epitope outside of the Wnt-binding motif. 32A9, similar to others, showed potent antitumor activity in vitro or in vivo [106].

## 6. Conclusions

It seems that some of the introduced immunotoxins have high potential for further investigations. For example, a dramatic decrease in the tumor volume of mice treated with anti-c-Met/PE38KDEL, humanized YP7-PE38, HN3-PE38, and HN3-mPE24 was observed. Furthermore, in spite of the clinical applicability of most immunotoxins based on the authors’ suggestions, the VB4-845 immunotoxin is the only one progressing in phase II and III trials. The major problem of overall immunotoxins is their short half-life [17], especially for those small in size, such as HN3. The vascular leak syndrome (VLS), which led to a major dose-limiting toxicity of immunotoxin therapy, is another key problem of the introduced immunotoxins [107]. Therefore, a low-dose, high-frequency treatment might be effective [48]. However, identifying the VLS-inducing motifs and their deletion or mutation as well as SMFv-PE38KDEL (mut 1) is one of the validated approaches. Another strategy to increase the efficiency of immunotoxins can be silencing T cell epitopes to further decrease immunogenicity [74] and combination with chemotherapy or immune suppression [75]. There are many tumor markers on HCC cells that have not been yet targeted, such as EGFR, FGFR, PDGFR, MUC1, and mesothelin. Furthermore, this review showed that several forms of truncated PE toxin were applied to produce immunotoxins against HCC, while different combinations of toxin and targeting moieties against HCC might have promising results.

## Figures and Tables

**Table 1:** HCC-specific tumor markers.

Tumor Marker	Type	Location	The Expression Pattern in Liver Cancer Tissues	Samples	Role	History of Targeting by Immunotoxin	Ref.
Glypican 3 (GPC3)	Onco-fetal	Cell surface	90%	133	Cell growth, development, differentiation, and migration	Yes (Y)	[74]
Epithelial cell adhesion molecule (EPCAM)	Overexpressed antigen	Cell surface	34.1%	132	Cell–cell adhesion, cell proliferation, tumorogenisity, metastasis	Y	[75]
Epidermal growth factor receptor (EGFR)	Overexpressed antigen	Cell surface	68%		Tumor cell proliferation, apoptosis, invasion, metastasis, and angiogenesis	Y	[76]
Fibroblast growth factor receptor (FGFR)	Overexpressed antigen	Cell surface	Nearly 50%	Five HCC cell lines §	Regulation of the tumor microenvironment and angiogenesis, morphology changes from epithelial to mesenchymal (EMT)	Y	[77]
Platelet-derived growth factor receptor (PDGFR-α and β)	Overexpressed antigen	Intracellular and Cell surface	68–73%	63	Blood vessel formation, regulation of cell growth and division, wound healing, and metastasis	Y	[78]
CD40	Overexpressed antigen	Cell surface	60%	45	Survival, proliferation, differentiation in B cells, chemotherapy resistance, and angiogenesis	Y	[79]
Mucin core protein 1 (MUC1)	Overexpressed antigen	Intracellular and Cell surface	70.8%	59 HCC and 37 CC	Preventing the pathogen from reaching the cell surface	Y	[80]
The C-X-C chemokine receptor type 7 (CXCR7)	Overexpressed antigen	Cell surface	-	Three cell lines ¶¶	Pro-angiogenic role in HCC	Y	[80]
Mesothelin	Tumor differentiation antigen, Overexpressed antigen	Cell surface	33% CCA and no expression in HCC	87	Cell adhesion	Y	[81]
TNF-related apoptosis-inducing ligand (TRAIL)	Overexpressed antigen	Cell surface	-	10	Lymphocyte cytotoxicity and the maintenance of immunological homeostasis in various tissues	Y	[82]
Tumor necrosis factor receptor superfamily member 12A (TNFRSF12A)	Overexpressed antigen	Cell surface	-	20	Tumor growth and metastasis	Y	[83]
Asialoglycoprotein receptor (ASGPR)	Overexpressed antigen	Cell surface	500,000 ASGPR/hepatocyte	-	Internalization of galactose (Gal)	Y	[66]
Receptor protein p230	Overexpressed antigen	Cell surface	-	-	Early tissues development	Y	[67]
E-cadherin	Overexpressed antigen	Cell surface	40%	37	Cell adhesion protein	Y	[84]
Axl receptor tyrosine kinase	Overexpressed antigen	Cell surface	-	Five HCC cell lines §	Tumor development and progression, differentiation, invasion, chemotherapy resistance	No (N)	[76,77]
γ-glutamyl transferase (GGT)	Overexpressed antigen	Cell surface	43.8%	120	Embryonic enzyme	N	[85]
Hypoxia-inducible factor (HIF)-1α	Overexpressed in HCC	Cell surface: Intracellular, Nucleoplasm, and Nuclear bodies	-	309	Tumor growth and metastasis	N	[86]
Ig gamma-1 chain C region	Overexpressed antigen	Cell surface and Secreted	-	25	-	N	[74]
β-catenin	Overexpressed antigen	Cell surface	78%	32	Generation/differentiation of many tissues	N	[87]
Frizzled receptors-2/7	Overexpressed antigen	Intracellular and Cell surface	95%	62	Mammalian hair follicle development	N	[88]

¶¶ HCCLM3, MHCC97-L, and SMMC-7721 § Huh7, HepG2, Hep3B/T2 HA22T/VGH, and HA59T/VGH cell lines.

**Table 2:** HCC-specific immunotoxins.

Label	Year	Targeting Moiety	Toxin Moiety	Receptor Type	Receptor Expression	Minimum Cell IC50	Ref.
hscFv25-TNFα	2000	scFv	TNFα	Unknown	---	---	[89]
hscFv25-mTNFα	2004	scFv	Mutant TNFα	Unknown	---	---	[90]
mut1	2007	SM5-1 single chain antibody (SMFv)	PE38KDEL	p230	Overexpressed	5.03 pmol/L	[95]
C1M	2011	scFv of anti- ASGPR	Melittin	ASGPR ¶	Overexpressed	<55 nmol/L	[91]
anti-c-Met/PE38KDEL	2013	scFv	PE38KDEL	c-Met	Overexpressed	150 pmol/L	[96]
VB4-845	2014	scFv	PE	EpCAM	Onco-fetal	<1 pmol/L	[97]
APE	2015	scFv	PE38KDEL	EpCAM	Onco-fetal	50 pmol/L	[20]
HN3-PE38	2015	VH domain	PE38	GPC3	Onco-fetal	0.28 nmol/L	[5]
YP7-PE38	2015	scFv	PE38	GPC3	Onco-fetal	1.59 nmol/L	[5]
HS20-PE38	2015	scFv	PE38	GPC3	Onco-fetal	---	[5]
YP7-PE38	2016	scFv	PE38	GPC3	Onco-fetal	7.8 ng/mL	[98]
Humanized YP7-PE38	2016	scFv	PE38	GPC3	Onco-fetal	28 ng/mL	[98]
YP9.1-PE38	2016	scFv	PE38	GPC3	Onco-fetal	2.9 ng/mL	[98]
Humanized YP9.1-PE38	2016	scFv	PE38	GPC3	Onco-fetal	77 ng/mL	[98]
HN3-mPE24	2017	VH domain	mPE24	GPC3	Onco-fetal	0.2 nM	[48]
HN3- HN3-mPE24	2017	VH domain	mPE24	GPC3	Onco-fetal	0.4 nM	[48]
HN3-T20	2019	VH domain	mPE24	GPC3	Onco-fetal	1.6 nM	[99]
HN3-ABD-T20	2019	VH domain	mPE24	GPC3	Onco-fetal	---	[99]

¶ Asialoglycoprotein receptor.

## Data Availability

Not applicable.
